# ROCK (RhoA/Rho Kinase) Activation in Atrial Fibrillation: Molecular Pathways and Clinical Implications

**DOI:** 10.2174/1573403X19666221117092951

**Published:** 2023-03-22

**Authors:** Riccardo Proietti, Andrea S. Giordani, Calò A. Lorenzo

**Affiliations:** 1 Liverpool Centre for Cardiovascular Science, University of Liverpool and Liverpool Heart & Chest Hospital, Liverpool, United Kingdom;; 2 Department of Cardiac, Thoracic, Vascular Sciences and Public Health, University of Padua, Padua, Italy;; 3 Department of Medicine (DIMED), Nephrology, Dialysis and Transplantation Unit, University of Padua and Azienda Ospedale Università di Padova, Padua, Italy

**Keywords:** Rho kinase, atrial fibrillation, calcium overload, myocardial remodelling, atrial fibrosis, oxidative stress

## Abstract

Among the complex mechanisms of AF pathogenesis, intracellular calcium overload and oxidative stress play a major role, both triggered by inflammatory processes. The additional basic event taking place in AF is atrial fibrotic remodeling, again triggered by oxidative stress, which is determined by connexins rearrangement and differentiation of fibroblasts into active collagen-secreting myofibroblasts. RhoA/ROCK system is the final pathway of a wide spectrum of molecular effectors such as Angiotensin II, platelet-derived growth factor, connective tissue growth factor and transforming growth factor β, that overall determine calcium dysregulation and pro-fibrotic remodeling. Both in experimental and clinical studies, RhoA/ROCK activation has been linked to superoxide ion production, fibrotic remodeling and connexins rearrangement, with important consequences for AF pathogenesis. ROCK pathway inhibition may therefore be a therapeutic or preventive target for special AF subgroups of patients.

## 1 INTRODUCTION

Atrial fibrillation (AF) is the most prevalent arrhythmia worldwide, and over the next few decades, its incidence is predicted to rise dramatically [1]. AF has a negative impact on quality of life, morbidity and mortality since it raises the risk of stroke, heart failure and dementia, determining a relevant healthcare burden [2]. AF is characterized by the loss of atrial muscle contractility and an erratic electrical activation, determined by initiating triggers, especially ectopic foci found inside the pulmonary veins, and aberrant atrial tissue fibrotic substrates. The etiology of AF is multifaceted, and the understanding of underlying molecular mechanisms is key in providing novel therapeutic strategies. Recently, the role of the Rho-associated coil-forming protein kinase (ROCK) protein family has emerged as key in multiple molecular pathways that can trigger and sustain AF development, both in terms of ion channel dysregulation and pro-fibrotic atrial remodeling [3]. ROCK is involved in the regulation of cytoskeletal reorganization by phosphorylation of the myosin light chain, which is its primary molecular target. Therefore, the ROCK pathway is involved in regulating endothelial migration, platelet activation, thrombosis, and oxidative stress as well as smooth muscle contraction. ROCK pathway has already shown to be involved in a variety of cardiovascular diseases, including arterial hypertension [4], stroke [5] and cardiovascular-renal remodelling [6], and its role in AF induction and perpetuation is recently under increasing investigation. Experimental studies on ROCK inhibitors show that it may be the target of therapeutic agents that, by facing ROCK-mediated cardiac remodelling induced by ischemic injury or hypertrophic stress, can potentially treat or prevent cardiovascular diseases [7, 8]. The aim of this review is to describe the physiological role of ROCK and its alterations during AF onset in experimental models, to summarize existing evidence on the ROCK role in AF pathogenesis and to address existing and possible future clinical implications.

## 2 MOLECULAR PATHOGENESIS OF ATRIAL FIBRILLATION: CALCIUM OVERLOAD AND ATRIAL FIBROSIS

The molecular mechanisms underpinning the development of AF can be outlined in two major processes; ion channel dysfunction and fibrotic remodeling. Firstly, electrical cellular remodelling in AF includes shortening of action potential duration and effective refractory period, which are caused by disturbances in ion channel activity [9]. Among the ion current alterations, those leading to intracellular calcium (Ca) concentration play a primary role in promoting AF onset [10]. Both focal ectopic (triggered) activity and reentry are promoted by Ca-handling anomalies, which contribute to AF initiation and maintenance. In fact, intra-cytoplasmic calcium overload reduces action potential duration and causes early after-depolarization (EAD) and delayed after-depolarization (DAD). Both EAD and DAD promote on the one side ectopic triggered activity of muscular sleeves around the pulmonary veins, and on the other side re-entry mechanisms in the atrial myocardium. Numerous studies have clarified that the dysfunction of ryanodine receptor type-2 (RyR2) determined either by the CaMKII (Ca/calmodulin-dependent protein kinase II) mediated hyper-phosphorylation or oxidation due to reactive oxygen species (ROS), is responsible for increased sarcoplasmic reticulum (SR) calcium leak and spontaneous calcium release from SR [11]. In turn, increased activity of the SR Ca-ATPase-2a (SERCA2a) or elevated intracellular sodium (Na) reduces the Ca-extrusion *via* the NCX1 (Na/Ca exchanger type 1) enhancing SR calcium overload [12]. In addition, calcium overload promotes L-type Ca-current (ICa, L)-dependent triggered calcium waves (TCW). In physiological conditions, calcium is released in the atrial cells following calcium sparks that occur at the cell boundary, which then diffuse to the cell interior. Conversely, when atrial cells are paced rapidly, the RyR2 receptors become more sensitized and generate calcium waves that propagate to the cell interior. TCW are supposed to be highly arrhythmogenic since by having a higher likelihood of producing wave breaks because of the short coupling intervals between paced beats. As a consequence, spontaneous calcium release events (SCaEs) and TCW activate a transient-inward current mediated by NCX (INCX- Na/Ca-exchanger type 1) resulting in DADs or EAD, depending on their timing relative to the atrial action potential. SR calcium overload and CaMKII hyperactivity can also cause by themselves TCWs and CaMKII-dependent dysregulation of I_Na,late_, producing proarrhythmic activity in atrial cardiomyocytes [13]. Interestingly, distinct molecular mechanisms are implicated in different clinical phenotypes of AF presentation. In paroxysmal AF, Ca overload appears to be mainly mediated by protein kinase A (PKA)-dependent hyper-phosphorylation of SERCA2a phospholamban leading to enhanced SR calcium uptake [14]. Furthermore, an enhanced overexpression of RyR2, in its open form due to a relative deficiency of Junctophilin 2, is reported to determine SCaE occurring in paroxysmal AF [15]. Conversely, in long-lasting AF, which seems to be predominant is the dysregulation of RyR2 mediated by CaMKII with a unique mechanism of auto-phosphorylation, which is enhanced by a rapid heart rate [16]. Oxidative stress is recognized to promote Ca-mediated triggers and the initiation of AF [17]. Several studies proved that reactive oxygen species (RSO), especially those that originated in the mitochondria, provoke the oxidation and subsequent activation of CAMKII leading to hyperphosphorylation of RYR2, with an arrhythmogenic effect [18], and that mitochondrial oxidative stress leads to increased SR calcium leak *via* oxidized RyR2 channels [19]. The second basic mechanism occurring in AF is atrial fibrotic remodeling. In fact, the understanding of AF pathogenesis has shifted from a rhythm disturbance towards a composite concept of atrial cardiomyopathy due to fibrosis arrhythmic substrate. Central to negative atrial remodeling is the transformation of silent fibroblasts into active pro-fibrotic collagen-secreting myofibroblasts. The activated myofibroblasts contribute actively to the deposition of extracellular matrix, consisting of procollagen and several glycoproteins including fibronectin, which promotes formation of a reactive fibrosis around myocardial cells [20]. A variety of cell membrane receptor systems promote the differentiation of fibroblast into secreting myofibroblasts, including Angiotensin-II (Ang II), platelet-derived growth factor, connective tissue growth factor, and transforming growth-factor β, all under control of ROCK signaling [21]. A prominent role appears to be played by the AT1R (Angiotensin II/angiotensin II type I) receptor, which leads to reactive species generation (ROS) and NLRP3-inflammasome formation and pro-fibrotic inflammatory signaling [22]. In addition, intracellular ROS production alters connexin function, either directly or *via* the activation of kinases like CaMKII or JNK. Connexins are the major components of atrial gap junctions, and Connexin40 (Cx40) especially determines the properties of intercellular conduction within atrial tissue [23]. Indeed, patients with paroxysmal AF show a reduced Cx40 expression and increased heterogeneity of the Cx40 distribution, while persistent AF patients show severe reductions of Cx40-immunostaining [24, 25]. It has been demonstrated that alterations in the relative amount and distribution of atrial connexin 40 and connexin 43 are influenced by inflammation levels [26]. In rat models, the ROCK pathway has been identified as a promoter of fibrosis, especially in the myocardium and particularly in a pro-inflammatory state such as acute ischemia [27]. In light of this, both electrical and fibrotic adverse remodeling appear as complex phenomena, with the ROCK pathway being primarily involved. Firstly, Rho signalling is recognized to have a role in the regulation of the cardiac conduction system: in animal studies, inhibition of the Rho pathway in transgenic mice (*via* increased expression of Rho GDIα, a GDP dissociation inhibitor specific for Rho family proteins), led to atrial arrhythmias and atrioventricular conduction defects [28]. Secondly, ROCK is an established mediator of inflammation, and its inhibition has been proven to lead to the resolution of acute inflammation [29]. Several studies have shown that an overall influence of inflammation exists in AF development [30-32]. Therefore, the interest in the role of the ROCK molecular pathway and AF is being extensively investigated.

## 3 RHO KINASE AND AF: MOLECULAR MECHANISMS

The Rho kinase (ROCK) protein family includes the RhoA/ROCK system, which plays an important role in a wide spectrum of physiological processes at a cellular signal transduction level [8]. In particular, ROCK is a serine/threonine kinase that functions as a downstream effector of RhoA, a small GTP-binding protein that controls a wide variety of signal transduction pathways. Protein kinases mediate which is probably the most universal and fundamental mechanism for the regulation of cellular protein function: protein phosphorylation [33]. Similarly to other Rho GTPases, ROCK protein superfamily proteins have a “molecular switch” role, cycling between a GTP-bound state, which is active, and a GDP-bound state, which corresponds to inactive [34]. Protein phosphorylation catalysed by protein kinases consists of the transfer of a phosphate group of ATP to a substrate, or in the binding of GTP as an effect of extracellular stimuli. ROCK activity is enhanced by the binding of the GTP-bound active form of RhoA [35]. ROCK1 (Rho kinase beta) and ROCK2 (Rho kinase alfa) are two isoforms of Rho-kinase that have different functions [36]: ROCK1 genes, located on chromosome 18, are expressed in circulating inflammatory cells, and ROCK2 genes, located on chromosome 2, are expressed in vascular smooth muscle cells (VSMCs). ROCK1 and ROCK2 share 92% homology in the kinase domains. The ROCK pathway has been shown to have a role in a variety of key cellular activities, including contraction, motility, proliferation, and apoptosis, which can lead to a variety of cardiovascular diseases [3]. Specifically, Rho-kinase mediates VSMCs hypercontraction, stimulates VSMCs proliferation and migration, and enhances inflammatory cells motility [3, 6]. ROCK also modulates cytoskeletal reorganization through the phosphorylation of myosin phosphatase [37], leading to an increase in myosin light chain phosphorylation promoting endothelial migration, platelet activation, thrombosis, and smooth muscle contraction [34]. The primary substrate of activated ROCK is Myosine phosphatase targeting subunit 1 (MYPT-1) in myosin light chain phosphatase (MLCP) [38]; this is relevant because high levels of the phosphorylated form of MYPT-1 can serve as a surrogate of the activation of ROCK in experimental models. The binding of Ang II and Endothelin 1, fibroblast growth factor (FGF) and transforming growth factor (TGF) and thrombin to their cell membrane receptors activates the ROCK-signaling pathway. Regarding Ang II, the first step in the activation process triggered by the binding to Ang II receptor is the mobilization of the Gα of the Gq protein, which activates the Rho-guanine exchange factors (RhoGEFs) to replace guanosine-50-diphosphate (GDP) with GTP. The GTP-Rho interaction with the RBD domain of Rho-Kinase leads to ROCK activation [39]. The activation of ROCK induces the activation of the NAD(P)H Oxidase, which is one of the most important enzymes responsible for superoxide ion production [40]. In fact, in the animal model, Higashi *et al.* have shown that Fasudil, an inhibitor of ROCK, prevents the overexpression of Angiotensin induction of NAD(P)H Oxidase [41]. Various *in vitro* studies proved that reactive oxygen species activate the ROCK pathway [42]. Oxidative stress induces ROCK activation and therefore entails the activation of extracellular signal-regulated kinase (ERK), the mitogen-activated protein kinase (MAPK) as well as the NFkB and PAI-2, which are directly involved in fibrosis and myocardial remodelling [43]. In addition, the oxidative stress induced by NAD(P)H Oxidase will directly activate the phospholipase C and increase inosithol3P and intracellular Ca, leading to a vicious cycle of intracellular calcium leak and AF triggering [44]. Rho-kinase can *per se* increase inflammation *via* the upregulation of pro-inflammatory cytokines, including IL-6, macrophage migration inhibitory factor and sphingosine-1-phosphate [45]. Similarly, the expression of Rho-kinase is accelerated by inflammatory stimuli, such as Ang II itself [46]. Rho-kinase also upregulates NAD(P)H oxidases and augments ANG II-induced ROS production [41]. Interestingly, experimental studies on mice have shown that pro-inflammatory states, such as hypercholesteraemic diets, determine on the one hand higher levels of ROCK activity in the cardiac tissue, and on the other significant reductions of antioxidant activity both in the heart and the serum [47]. ROCK is only one of the many kinase families that are under investigation for their role in AF pathogenesis. The Rho GTPase Rac1 also has been identified as a contributor to the pathogenesis of AF: in studies on mice, those with chronic cardiac overexpression of Rac1 developed AF [48]. Similarly to ROCK, studies have shown that Tec kinases are involved in AF induction and maintenance by sustaining cellular inflammatory processes [33], such as the activation of NLRP3- inflammasome in cardiomyocytes, which might contribute to the progression of atrial remodelling [49]. This emphasises the mutual interaction of cellular phosphorylation transduction signalling and inflammatory molecular pathways.

## 4 RHO KINASE ROLE IN AF: EXPERIMENTAL AND CLINICAL EVIDENCE

Despite solid molecular evidence for ROCK playing a significant role in AF pathogenesis, only a few experimental and clinical studies have specifically investigated the role of ROCK pathway activation in AF in recent years. A list of the most relevant experimental studies specifically exploring the role of the ROCK pathway in AF is reported in Table **1**. Given the established role of ROCK in the pro-fibrotic remodelling of different organs and the beneficial role of its inhibition [50-52], experimental studies have addressed the role of ROCK signalling in atrial fibrosis. Among ROCK inhibitors, two major agents have been identified, Fasudil and Y-27632: both target ROCK ATP-dependent kinase domains and are non-isoform-selective inhibitors, *i.e*. they equally inhibit both ROCK1 and ROCK2. Fasudil is more widely studied because is best available for long-term use *in vivo* and is the only agent approved for use in humans. Chen *et al.* conducted a study on rat models of type 2 diabetes to test the efficacy of Fasudil hydrochloride on atrial fibrosis, quantified as collagen density at Masson staining. Compared to controls, rats receiving Fasudil showed reduced atrial fibrosis, together with reduced levels of both mRNA and protein levels of RhoA, ROCK1, ROCK2, type-I and type-III procollagen-1. The results suggested that ROCK is actively involved in atrial fibrosis, and that its inhibition reduces atrial negative remodelling *via* the ROCK pathway in the experimental models [53]. Moreover, Guan *et al.* showed the beneficial effect of Fasudil on cardiomyopathy in experimental model of diabetic rats: long-term Fasudil administration had a cardio-protective effect in hemodynamic (low left ventricular diastolic pressure), biochemical (lower levels of circulating Lactate Dehydrogenase and Creatine Phosphokinase, as expression of myocardial damage) and histopathologic terms (less cardiac hypertrophy) [54]. Notably, these effects were similar to those of captopril, an established cardio-protective drug effective in diabetic cardiomyopathy. Moreover, the activity of superoxide dismutase, a potent anti-oxidant enzyme, was markedly improved in rats treated with Fasudil, similar to those treated with captopril, leading to a lower degree of cardiomyocytes apoptosis, marking an important role of Fasudil as oxidative stress antagonist. In a similar way, Zhou *et al.* [55] demonstrated that Fasudil had a positive effect on myocardial fibrosis in type 2 diabetes murine models through the inhibition of the Jc-Jun NH_2_-terminal kinase (JNK) and TGFβ/Smad pathways: in treated mice, a reduction of collagen fibres deposition in the interstitial spaces among cardiomyocytes was observed by transmission electron microscopy. Apart from its role in atrial fibrosis, ROCK signaling may trigger AF by altering intercellular electrophysiological connections, particularly *via* gap junctions and connexins remodelling. In an animal model of chronic kidney disease, Qiu *et al.* [56] showed that reduced expression of Cx40 and increased expression of both Cx43 and N-cadherin leads to proarrhythmic cardiac remodeling and to an increased susceptibility to AF induction. In particular, rat models with kidney disease showed increased rates of AF inducibility by transesophageal atrial pacing, and longer duration of AF episodes, compared to controls. Apart from connexins remodelling, these proarrhythmic changes were attributed to the activation of NLRP3 (NOD-, LRR- and pyrin domain-containing protein 3) inflammasome signalling, remarking the role of inflammation in AF development. Notably, in the same study, Qiu *et al.* reported that the Cxs changes found were mediated by Ang II through AT1R activation by a protein of the Rho GTPase superfamily [56]. This finding raises questions as to the exact role of the ROCK pathway of Ang II transduction in the molecular mechanisms linked to AF. More recently, Yongqing *et al.* [57], examining human left atrial appendages from 40 patients undergoing cardiac surgery, found that, when AF was present, an overexpression of ROCK, its metabolic downstream mediator myosin phosphatase target subunit-1 (MYPT-1), and of Cx40 was observed. This is of importance since Cx 40 is an important component of gap junction proteins in the human heart, whose alteration may have an impact on the coordination of action potential in the atrial myocardium. In human patients with end-stage renal disease (ESRD) on dialysis and with permanent AF, Calò *et al.* [58] documented increased MYPT-1 phosphorylation and Cx40 levels compared to either patient with ESRD on dialysis without AF or healthy controls. Dialysis patients are good candidates for studying the interaction of ROCK and AF *in vivo* since AF is the most frequent arrhythmia in dialysis ESRD patients that also presented increased MYPT-1phosphorylation, which correlated with left ventricular mass [59]. In this small cohort of 20 ERDS patients, those without AF did not have increased Cx40, and only AF patients have increased levels of MYPT-1 phosphorylation and Cx40, with a positive linear correlation. In the same study, treatment with Fausidil led to a decrease in levels of phospho-MYPT-1, with a subsequent reduction of the Cx40 protein expression. This suggests that Cx40 may be a downstream target of the ROCK pathway activation. Additional support for this hypothesis might be found *via* the ERK pathway control of Cxs remodeling as ERK is one of the downstream pathways of MAPK, which is upstream regulated by ROCK. Finally, in 2019 Düzen *et al*. [60] explored gene expression of ROCK genes in circulating leukocytes of patients with nonvalvular AF: compared to healthy controls, individuals with AF had a significant marked increase in the ROCK1 and ROCK2 gene expressions. These data show that the ROCK pathway may contribute to the pathogenesis of AF through activated leukocytes, *via* promoting the inflammatory cascade.

## CONCLUSION

In the complex pathogenesis of AF, ROCK signalling plays a major role. ROCK mediates the effect of inflammatory triggers by altering calcium channel ionic currents, altering connexion gap junction electrical connections, and mediating pro-fibrotic cardiac remodelling. Robust experimental and clinical evidence exists on the role of the ROCK pathway in enhancing AF onset and perpetuation, along with the beneficial effects of its inhibition. Cardiac-specific regulation of ROCK pathways could be a promising therapeutic, or even prophylactic, strategy for certain populations of AF patients.

## Figures and Tables

**Table 1:** Experimental and clinical studies specifically addressing the role of ROCK signalling in AF.

**Author**	**Year**	**Journal**	**Type of Study**	**Use of ROCK Inhibitors**	**Main Subject**
Wei *et al*.	2004	FASEB	Animal	No	Role of Rho GTPases in regulating AV conduction
Zhou *et al*.	2011	Acta Pharmacol Sin	Animal	Yes	ROCK signalling in myocardial fibrosis in a rat model of type 2 diabetes
Chen *et al*.	2014	Exp Ther Med	Animal	Yes	ROCK pathway in atrial fibrosis of type 2 diabetes in rats
Calò *et al*.	2016	Life Sci	Human	Yes	ROCK activity in mononuclear cells of chronic kidney disease patients
Yongqing *et al*.	2018	Heart Surg Forum	Human	No	ROCK expression in left atrial appendage in AF patients
Düzen *et al*.	2019	Exp Ther Med	Human	No	ROCK gene expressions in circulating leucocytes of patients with non-valvular AF
Calò *et al*.	2020	J Clin Med	Human	Yes	Assessment of mononuclear cells’ MYPT-1 phosphorylation, Cx40 expression, and effect of Fasudil in dialysis patients with AF

## LIST OF ABBREVIATIONS

ESRD: End-Stage Renal Disease
MLCP: Myosin Light Chain Phosphatase
VSMCS: Vascular Smooth Muscle Cells
ROCK: Rho Kinase
MAPK: Mitogen-Activated Protein Kinase

## References

[1] Krijthe B.P., Kunst A., Benjamin E.J. (2013). Projections on the number of individuals with atrial fibrillation in the European Union, from 2000 to 2060.. Eur. Heart J..

[2] Proietti M., Laroche C., Nieuwlaat R. (2018). Increased burden of comorbidities and risk of cardiovascular death in atrial fibrillation patients in Europe over ten years: A comparison between EORP-AF pilot and EHS-AF registries.. Eur. J. Intern. Med..

[3] Shimokawa H., Sunamura S., Satoh K. (2016). RhoA/Rho-kinase in the cardiovascular system.. Circ. Res..

[4] Masumoto A., Hirooka Y., Shimokawa H., Hironaga K., Setoguchi S., Takeshita A. (2001). Possible involvement of Rho-kinase in the pathogenesis of hypertension in humans.. Hypertens.

[5] Shibuya M., Hirai S., Seto M., Satoh S., Ohtomo E. (2005). Effects of fasudil in acute ischemic stroke: Results of a prospective placebo-controlled double-blind trial.. J. Neurol. Sci..

[6] Seccia T.M., Rigato M., Ravarotto V., Calò L.A. (2020). ROCK (RhoA/Rho Kinase) in cardiovascular–renal pathophysiology: A review of new advancements.. J. Clin. Med..

[7] Surma M., Wei L., Shi J. (2011). Rho kinase as a therapeutic target in cardiovascular disease.. Future Cardiol..

[8] Shi J., Wei L. (2013). Rho kinases in cardiovascular physiology and pathophysiology: The effect of fasudil.. J. Cardiovasc. Pharmacol..

[9] Dobrev D., Ravens U. (2003). Remodeling of cardiomyocyte ion channels in human atrial fibrillation.. Basic Res. Cardiol..

[10] Wang X., Chen X., Dobrev D., Li N. (2021). The crosstalk between cardiomyocyte calcium and inflammasome signaling pathways in atrial fibrillation.. Pflugers Arch..

[11] Williams G.S.B., Chikando A.C., Tuan H.T.M., Sobie E.A., Lederer W.J., Jafri M.S. (2011). Dynamics of calcium sparks and calcium leak in the heart.. Biophys. J..

[12] Dridi H., Kushnir A., Zalk R., Yuan Q., Melville Z., Marks A.R. (2020). Intracellular calcium leak in heart failure and atrial fibrillation: A unifying mechanism and therapeutic target.. Nat. Rev. Cardiol..

[13] Heijman J., Voigt N., Wehrens X.H.T., Dobrev D. (2014). Calcium dysregulation in atrial fibrillation: The role of CaMKII.. Front. Pharmacol..

[14] Vest J.A., Wehrens X.H.T., Reiken S.R. (2005). Defective cardiac ryanodine receptor regulation during atrial fibrillation.. Circulation.

[15] Beavers D.L., Wang W., Ather S. (2013). Mutation E169K in junctophilin-2 causes atrial fibrillation due to impaired RyR2 stabilization.. J. Am. Coll. Cardiol..

[16] Wehrens X.H.T. (2011). CaMKII regulation of the cardiac ryanodine receptor and SR calcium release.. Heart Rhythm.

[17] Karam B.S., Chavez-Moreno A., Koh W., Akar J.G., Akar F.G. (2017). Oxidative stress and inflammation as central mediators of atrial fibrillation in obesity and diabetes.. Cardiovasc. Diabetol..

[18] Shan J., Xie W., Betzenhauser M. (2012). Calcium leak through ryanodine receptors leads to atrial fibrillation in 3 mouse models of catecholaminergic polymorphic ventricular tachycardia.. Circ. Res..

[19] Joseph L.C., Barca E., Subramanyam P. (2016). Inhibition of NAPDH Oxidase 2 (NOX2) prevents oxidative stress and mitochondrial abnormalities caused by saturated fat in cardiomyocytes.. PLoS One.

[20] Sohns C., Marrouche N.F. (2020). Atrial fibrillation and cardiac fibrosis.. Eur. Heart J..

[21] Nattel S. (2017). Molecular and cellular mechanisms of atrial fibrosis in atrial fibrillation.. JACC Clin. Electrophysiol..

[22] Harada M., Nattel S. (2021). Implications of inflammation and fibrosis in atrial fibrillation pathophysiology.. Card. Electrophysiol. Clin..

[23] Lin X., Gemel J., Glass A., Zemlin C.W., Beyer E.C., Veenstra R.D. (2010). Connexin40 and connexin43 determine gating properties of atrial gap junction channels.. J. Mol. Cell. Cardiol..

[24] Gemel J., Levy A.E., Simon A.R. (2014). Connexin40 abnormalities and atrial fibrillation in the human heart.. J. Mol. Cell. Cardiol..

[25] Hauer R.N.W., Groenewegen W.A., Firouzi M., Ramanna H., Jongsma H.J. (2006). Cx40 polymorphism in human atrial fibrillation.. Adv. Cardiol..

[26] Ryu K., Li L., Khrestian C.M. (2007). Effects of sterile pericarditis on connexins 40 and 43 in the atria: Correlation with abnormal conduction and atrial arrhythmias.. Am. J. Physiol. Heart Circ. Physiol..

[27] Gao H.C., Zhao H., Zhang W.Q., Li Y.Q., Ren L.Q. (2013). The role of the Rho/Rock signaling pathway in the pathogenesis of acute ischemic myocardial fibrosis in rat models.. Exp. Ther. Med..

[28] Wei L., Taffet G.E., Khoury D.S. (2004). Disruption of Rho signaling results in progressive atrioventricular conduction defects while ventricular function remains preserved.. FASEB J..

[29] Galvão I., Athayde R.M., Perez D.A. (2019). ROCK inhibition drives resolution of acute inflammation by enhancing neutrophil apoptosis.. Cells.

[30] Zhou X., Dudley S.C. (2020). Evidence for inflammation as a driver of atrial fibrillation.. Front. Cardiovasc. Med..

[31] Korantzopoulos P., Letsas K.P., Tse G., Fragakis N., Goudis C.A., Liu T. (2018). Inflammation and atrial fibrillation: A comprehensive review.. J. Arrhythm..

[32] Hu Y.F., Chen Y.J., Lin Y.J., Chen S.A. (2015). Inflammation and the pathogenesis of atrial fibrillation.. Nat. Rev. Cardiol..

[33] Yin Z., Zou Y., Wang D. (2022). Regulation of the Tec family of non-receptor tyrosine kinases in cardiovascular disease.. Cell Death Discov..

[34] Rolfe B., Worth N., World C., Campbell J., Campbell G. (2005). Rho and vascular disease.. Atherosclerosis.

[35] Matsui T., Amano M., Yamamoto T. (1996). Rho-associated kinase, a novel serine/threonine kinase, as a putative target for small GTP binding protein Rho.. EMBO J..

[36] Nakagawa O., Fujisawa K., Ishizaki T., Saito Y., Nakao K., Narumiya S. (1996). ROCK-I and ROCK-II, two isoforms of Rho-associated coiled-coil forming protein serine/threonine kinase in mice.. FEBS Lett..

[37] Shimokawa H., Takeshita A. (2005). Rho-kinase is an important therapeutic target in cardiovascular medicine.. Arterioscler. Thromb. Vasc. Biol..

[38] Kimura K., Ito M., Amano M. (1996). Regulation of myosin phosphatase by Rho and Rho-associated kinase (Rho-Kinase).. Science.

[39] Tagawa M., Nakamura Y., Okura Y. (2019). Successful treatment of acute fulminant eosinophilic myocarditis in a patient with ulcerative colitis using steroid therapy and percutaneous cardiopulmonary support.. Intern. Med..

[40] Schröder K. (2019). NADPH oxidase-derived reactive oxygen species: Dosis facit venenum.. Exp. Physiol..

[41] Higashi M., Shimokawa H., Hattori T. (2003). Long-term inhibition of Rho-kinase suppresses angiotensin II-induced cardiovascular hypertrophy in rats in vivo: Effect on endothelial NAD(P)H oxidase system.. Circ. Res..

[42] Jin L., Ying Z., Webb R.C. (2004). Activation of Rho/Rho kinase signaling pathway by reactive oxygen species in rat aorta.. Am. J. Physiol. Heart Circ. Physiol..

[43] (2010). Juan-Zhang , Bian HJ, Li XX, et al.ERK-MAPK signaling opposes rho-kinase to reduce cardiomyocyte apoptosis in heart ischemic preconditioning.. Mol. Med..

[44] Hu Q., Zheng G., Zweier J.L., Deshpande S., Irani K., Ziegelstein R.C. (2000). NADPH oxidase activation increases the sensitivity of intracellular Ca2+ stores to inositol 1,4,5-trisphosphate in human endothelial cells.. J. Biol. Chem..

[45] Radeff J.M., Nagy Z., Stern P.H. (2004). Rho and Rho kinase are involved in parathyroid hormone-stimulated protein kinase C alpha translocation and IL-6 promoter activity in osteoblastic cells.. J. Bone Miner. Res..

[46] Hiroki J., Shimokawa H., Higashi M. (2004). Inflammatory stimuli upregulate Rho-kinase in human coronary vascular smooth muscle cells.. J. Mol. Cell. Cardiol..

[47] Ma Z., Zhang J., Ji E., Cao G., Li G., Chu L. (2011). Rho kinase inhibition by fasudil exerts antioxidant effects in hypercholesterolemic rats.. Clin. Exp. Pharmacol. Physiol..

[48] Adam O., Frost G., Custodis F. (2007). Role of Rac1 GTPase activation in atrial fibrillation.. J. Am. Coll. Cardiol..

[49] Yao C., Veleva T., Scott L. (2018). Enhanced cardiomyocyte NLRP3 inflammasome signaling promotes atrial fibrillation.. Circulation.

[50] Kikuchi Y., Yamada M., Imakiire T. (2007). A Rho-kinase inhibitor, fasudil, prevents development of diabetes and nephropathy in insulin-resistant diabetic rats.. J. Endocrinol..

[51] Kolavennu V., Zeng L., Peng H., Wang Y., Danesh F.R. (2008). Targeting of RhoA/ROCK signaling ameliorates progression of diabetic nephropathy independent of glucose control.. Diabetes.

[52] Jiang C., Huang H., Liu J., Wang Y., Lu Z., Xu Z. (2012). Fasudil, a Rho-kinase inhibitor, attenuates bleomycin-induced pulmonary fibrosis in mice.. Int. J. Mol. Sci..

[53] Chen J., Li Q., Dong R., Gao H., Peng H., Wu Y. (2014). The effect of the Ras homolog gene family (Rho), member A/Rho associated coiled-coil forming protein kinase pathway in atrial fibrosis of type 2 diabetes in rats.. Exp. Ther. Med..

[54] Guan S. (2012). Long-term administration of Fasudil improves cardiomyopathy in streptozotocin-induced diabetic rats.. Food Chem. Toxicol..

[55] Zhou H., Li Y., Wang M. (2011). Involvement of RhoA/ROCK in myocardial fibrosis in a rat model of type 2 diabetes.. Acta Pharmacol. Sin..

[56] Qiu H., Ji C., Liu W. (2018). Chronic kidney disease increases atrial fibrillation inducibility: Involvement of inflammation, atrial fibrosis, and connexins.. Front. Physiol..

[57] Chen Y, Su F, Han J, Jiao P, Guo W. (2018). Expression of Rho kinase and its mechanism in the left atrial appendage in patients with atrial fibrillation.. Heart Surg Forum.

[58] Calò L.A., Ravarotto V., Bertoldi G. (2020). Rho kinase activity, connexin 40, and atrial fibrillation: Mechanistic insights from end-stage renal disease on dialysis patients.. J. Clin. Med..

[59] Calò L.A., Vertolli U., Pagnin E. (2016). Increased rho kinase activity in mononuclear cells of dialysis and stage 3–4 chronic kidney disease patients with left ventricular hypertrophy: Cardiovascular risk implications.. Life Sci..

[60] Düzen I.V., Yavuz F., Vuruskan E. (2019). Investigation of leukocyte RHO/ROCK gene expressions in patients with non-valvular atrial fibrillation.. Exp. Ther. Med..

